# Assessing the Capacity of Ecosystems to Supply Ecosystem Services Using Remote Sensing and An Ecosystem Accounting Approach

**DOI:** 10.1007/s00267-018-1110-x

**Published:** 2018-09-28

**Authors:** Leonardo Vargas, Louise Willemen, Lars Hein

**Affiliations:** 1 Environmental Systems Analysis Group, Wageningen University and Research, PO Box 47, 6700 AA Wageningen, The Netherlands; 2 Faculty of Geo-Information Science and Earth Observation (ITC), University of Twente, PO Box 217, 7500 AE Enschede, The Netherlands; 3 Environmental Systems Analysis Group, Wageningen University and Research, PO Box 47, 6700 AA Wageningen, The Netherlands

**Keywords:** Ecosystem capacity, Net primary productivity, Colombia, Ecosystem change, Biomass, Orinoco river basin

## Abstract

Ecosystems contribute to economic development through the supply of ecosystem services such as food and fresh water. Information on ecosystems and their services is required to support policy making, but this information is not captured in economic statistics. Ecosystem accounting has been developed to integrate ecosystems and ecosystem services into national accounts. Ecosystem accounting includes the compilation of an ecosystem services supply and use account, which reflects actual flows of ecosystem services, and the ecosystem capacity account, which reflects the capacity of ecosystems to sustainably supply ecosystem services. A capacity assessment requires detailed data on ecosystem processes, which are often not available over large scales. In this study, we examined how net primary productivity derived from remote sensing can be used as an indicator to assess changes in the capacity of ecosystems to supply services. We examine the spatial and temporal patterns in this capacity for the Orinoco river basin from 2001 to 2014. Specifically, we analyze the capacity of six types of ecosystems to supply timber, pastures for grazing cattle, oil palm fresh fruit bunches and to sequester carbon. We compared ecosystem capacities with the level of ecosystem service supply to assess a sustainable use of ecosystems. Our study provides insights on how the capacity of ecosystems can be quantified using remote sensing data in the context of ecosystem accounting. Ecosystem capacity indicators indicate ecosystems change and harvesting-regeneration patterns which are important for the design and monitoring of sustainable management regimes for ecosystems.

## Introduction

Ecosystems provide a wide variety of ecosystem services essential for human survival, including the supply of food, the control of diseases and the regulation of floods (Carpenter et al. 2009; De Groot et al. 2002). Nevertheless, ecosystems have been unsustainably changing for decades as a consequence of increasing economic activities such as agriculture and industry (Foley et al. 2005; Steffen et al. 2015).The design and implementation of policies aiming to decrease unsustainable changes of ecosystems are constrained by a lack of policy relevant information (Daily et al. 2009; Tallis and Polasky 2009). Particularly, because international economic monitoring systems that compile policy relevant economic information such as the System of National Accounts (SNA) do not include sufficient environmental information required to monitor changes in ecosystems (United Nations et al. 2009). International efforts to develop a monitoring system that integrates economic and environmental information led to the development of the System of Environmental Economic Accounting Central-Framework (SEEA-CF), as an international standard integrated monitoring system (United Nations et al. 2014a). The SEEA-CF is complemented by the publication of the System of Environmental-Economic Accounting-Experimental Ecosystem Accounting (SEEA-EEA) to assess changes in ecosystems and the flow of ecosystem services, accounting for changes in stock and flows consistent with the SEEA-CF model (United Nations et al. 2014a; 2015). A key innovation in ecosystem accounting is the inclusion and guidance on the spatially explicitly measurement of stocks by assessing changes ecosystems in terms of extent, condition and capacity to supply ecosystem services, and the measurement of flows of ecosystem services (United Nations et al. 2014b). Whereas extent reflects changes in ecosystem’s size and location, condition reflects changes in its quality, and capacity reflect changes in the ability of an ecosystem to generate ecosystem services as a function of changes in extent and condition. In practical terms, two aspects can be distinguished from the occurrence of an ecosystem service; capacity and flow, where capacity is the long term ecosystem’s potential to sustainably generate an ecosystem service, and flow is the actual use of the service (Schröter et al. 2014). A clear distinction between capacity and flow is important because the generation of some ecosystem services involves harvest-regeneration patterns and for some ecosystem the generation of ecosystem services can be above its capacity. This is important to assess the overall sustainability of the human activities in such ecosystem. The concept of capacity can be used to assess a sustainable use of ecosystems, as capacity reflects the ability of an ecosystem to sustainably supply a service under current ecosystem condition and uses at the highest yield or use level (Hein et al. 2016; United Nations et al. 2017). Here, the supply of ecosystem services is sustainable when the supply of an ecosystem service does not negatively affect the future supply of the same or other ecosystem services from that ecosystem. Current ecosystem condition means that the capacity is defined as it is now, neglecting alternative uses and independently from normative and historical baseline reference conditions (Hein et al. 2016). Therefore, by quantifying the capacity of an ecosystem to supply ecosystem services, the maximum amount of ecosystem services that can be supplied in a sustainable way is defined.

Different spatially explicit methods can be used to assess the capacity of an ecosystem to supply ecosystem services, including biophysical models, static land cover-based look-up tables, remote sensing and direct measurements (Bagstad et al. 2013; Schröter et al. 2015; Willemen et al. 2015). Direct measurements are desirable (e.g. by harvesting and measuring pasture biomass to assess grazing capacity) but unrealistic for large areas. Land cover, land use data and ecosystem services biophysical models are combined using software modelling tools such as the Integrated Valuation of Ecosystem services and Trade-offs (InVEST) (Sharp et al. 2015) and the Artificial Intelligence for Ecosystem Services (ARIES) (Villa et al. 2014). In addition to these combination of methods, experts knowledge and statistic data are combined using a simple modelling tool as the matrix method (Burkhard et al. 2009; 2014). Because of requiring reliable and diverse input data, these modelling tools typically assess ecosystem services at one point in time. Satellite remote sensing has the ability to observe large areas offering an opportunity to overcome or complement extensive field surveys (Andrew et al. 2014; Crossman et al. 2012). Remote sensing has increasingly been used to support ecosystem services assessments in the last decade, the repeating observations have been delivering often freely accessible spectral information to monitor key aspects of ecosystems including primary productivity, carbon, nitrogen and water cycles (Andrew et al. 2014; Ayanu et al. 2012). Remote sensing has the potential to cover large areas when direct measurements are not practically implementable and spatial and temporal data for biophysical models is lacking. Because ecosystem accounting requires environmental data, spatially explicit, repeatable and accessible, suitable to assess large areas in various accounting periods, this study explored the use of remote sensed data to assess the capacity of an area to sustainable deliver ecosystem services.

The objective of this study is to explore if and how remote sensing spectral information can be used to assess the capacity of ecosystems to supply ecosystem services following the ecosystem accounting guidelines. Specifically, we use Net Primary Productivity (NPP) information from Moderate Resolution Imaging Spectroradiometer (MODIS) combined with additional ecosystem services data to analyse changes in the capacity of ecosystems to supply ecosystem services over time and space in the Orinoco river basin. We selected six ecosystems; forest, oil palm plantations, grassland, savannah, woody savannah and mixed ecosystems that supply the following ecosystem services: oil palm fresh fruit bunches (FFB), timber, pastures for cattle grazing and carbon sequestration. We analysed the spatial and temporal patterns in capacity, and we compared the capacity of these selected ecosystems with the supply of ecosystem services. We selected the Colombian side of the Orinoco River Basin as case study area because this area is one of the most pristine river basins of South America, while the river basin is at the same time witnessing fast degradation of ecosystems driven by economic development (Etter et al. 2010).

## Methods

### The Orinoco River Basin

**Fig. 1:**
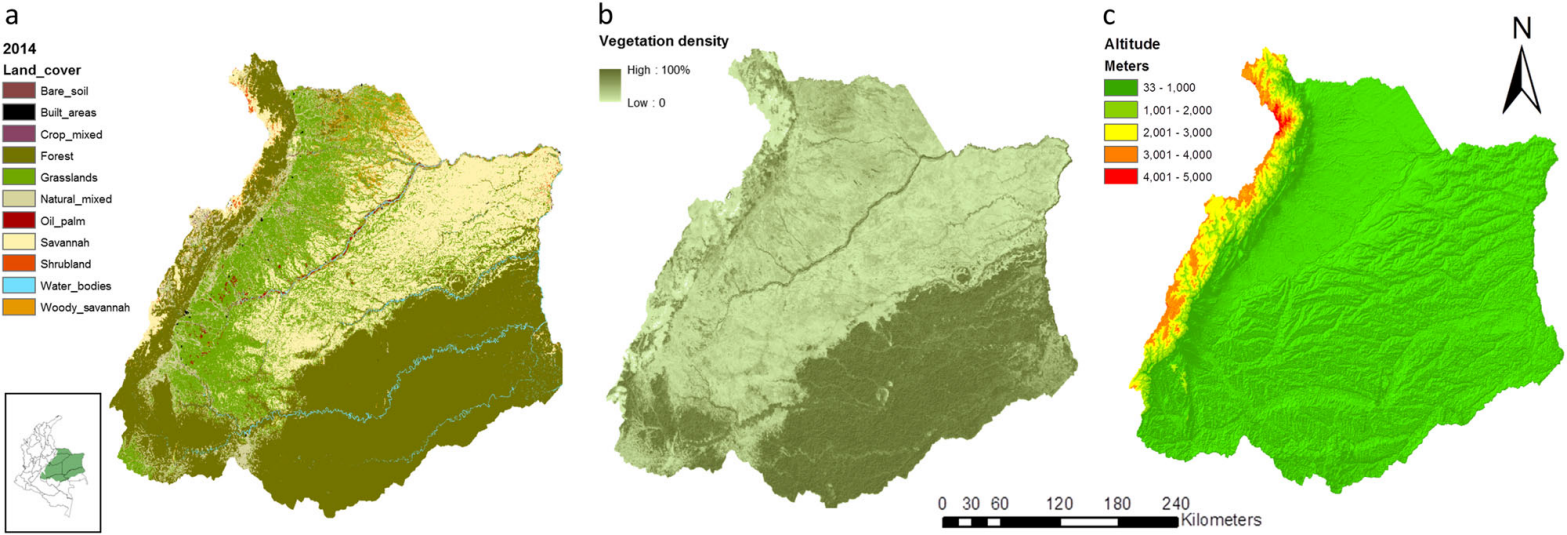
Maps showing in **a** the geographical boundaries of the Orinoco river basin in Colombia defining the EAA, and the EA based on the MODIS land cover product, **b** Vegetation density based on MODIS (MOD44B), and **c** Altitude based on digital elevation model (Global Multiresolution Terrain Elevation Data)

The Orinoco is a transboundary river basin covering 655,000 km^2^ in Venezuela and 345,000 km^2^ in Colombia (Wolf et al. 1999), see Fig. 1. Our study focuses on the Colombian part of the river basin covering the northern Andes mountains, the Guyana Shield, floodplains between the Orinoco and Amazon river basins, and high and low plains in the east. The basin is characterized by diverse ecosystems including páramos and cloud forests in the Andes mountains, natural savannah in the low plains and Amazon tropical rainforests (Lasso et al. 2010). The average annual temperature varies from below 0 °C in the mountains to 38 °C in the eastern plains, while the annual precipitation varies from 4000 mm on the eastern slopes of the cordillera to 1500 mm in the eastern plains (Llanos) (Lasso et al. 2010; León 2005). The area is one of the most pristine river basins of South America but witnesses fast changes in many ecosystems, driven by economic development (Etter et al. 2010). Land cover and land use transformations occur with the introduction of crops (e.g. oil palm, rice, soy) and improved grass species allowing the intensification of livestock production (Benavides 2010).

### Ecosystem Accounting Units

Ecosystem accounting uses three spatial units to organize information: ecosystem accounting areas (EAA), ecosystem assets (EA) and basic spatial units (BSU) (United Nations et al. 2017). EAA are large spatial areas defined by fixed and relative stable boundaries such as environmental management areas or administrative boundaries. In this study, we define the EAA by the boundaries of the Colombian Orinoco River Basin as mapped by the Colombian Instituto de Investigación de Recursos Biológicos Alexander von Humboldt (Romero-Ruiz et al. 2012). EA are spatial areas that form the conceptual base for accounting and where relevant statistics are integrated. The EA represent contiguous areas covering a specific type of ecosystem (e.g. forests, savannahs). Ecosystem accounting recommends the land cover classification presented in the SEEA-CF as a starting point to define EA (United Nations et al. 2014b). In our study, we use the International Geosphere-Biosphere Programme classification (IGBP) land cover classification as a starting point to define EA. The IGBP classification is included in the MODIS MCD12Q1 land cover product and describes 17 land cover types with an overall accuracy about 75% correctly classified (Friedl et al. 2010; Loveland and Belward 1997). We use the MODIS MCD12Q1 product because it provides annual land cover data that coincides with the standard length of the accounting period in ecosystem accounting: one year. To simplify the land cover map, we merge the five IGBP forest classes (evergreen needle leaf, deciduous needle leaf, evergreen broadleaf, deciduous broadleaf, mixed forest) into one forest class, we merge closed and open shrubland into one class, and water and permanent wetlands into one class. Hence, we reduce the 17 IGBP land cover types to 11 merged land cover types (Fig. 1a). The reclassification is needed as we are not able to differentiate ecosystem services supply between more than these 11 land cover classes, given the scale at which we work and the data availability for this area.

Of the 11 grouped land cover types, only six land cover types (forest, grassland, savannah, woody savannah, natural mixed and oil palm plantations) were relevant for the selected ecosystem services: oil palm FFB, grazing pastures for cattle, timber and carbon sequestration. These ecosystem services are included in this study because of their importance for economic development as well as their implications for ecosystem change. We link the selected ecosystem services with the six EA: oil palm FFB are supplied by oil palm plantations, grazing pastures are supplied by four ecosystems: grassland, savannah, woody savannah and natural mixed ecosystem (which are pastures and trees), and timber is supplied by forest ecosystem (see Supplemental Materials for more details on ecosystem services and EA). The accounting unit BSU is a small spatial area typically formed by grid tessellations (e.g. squares of 1 ha), like cadastral units or remote sensing pixels. In this study, we define BSU by pixels from the MODIS land cover product which are 21.4 hectare in size (463.3 m by 463.3 m in the study area).

### Assessing the Capacity of Ecosystems to Supply Ecosystem Services

#### Net primary productivity as an indicator of ecosystem capacities

To assess the capacity of ecosystems to supply ecosystem services, appropriate indicators need to be selected and quantified. Such indicators should be sensitive to changes in ecosystem condition and extent, and reflect changes in the future generation of ecosystem services. Ecosystem functioning indicators such as Net Primary Productivity (NPP) have been used in earlier assessments of ecosystem change and ecosystem services supply (Costanza et al. 2007; van Oudenhoven et al. 2012). NPP is the net carbon gain by plants after respiration, including all new plants biomass, soluble organic compounds secreted into the environment, carbon transfers to microbes in the root systems and volatile emissions from leaf tissues (Clark et al. 2001). We selected NPP as an indicator of the capacity of ecosystems to supply ecosystem services because of two aspects. First, NPP is sensitive to changes in ecosystem condition, driven by abiotic (e.g. light, temperature, precipitation, evapotranspiration, nutrients) and biotic (vegetation structure, biodiversity, herbivorous consumption) factors (Knapp et al. 2014). Second, all terrestrial ecosystems depends on NPP through plant photosynthesis to obtain energy and carbon, essential for the generation of ecosystem services (Chapin et al. 2011).

#### MODIS as a data source to derive NPP

To assess the capacity of ecosystems to supply ecosystem services, spatially explicit information was needed. We used annual accumulated spatially explicit NPP derived from the MODIS MOD17A3 for the time period between 2001 to 2014. MODIS (MOD17A3) provides high quality globally validated modelled NPP estimates based on the Monteith and Moss (1977) radiance use efficiency algorithm (Running and Zhao 2015). NPP depends on the amount of light reaching vegetation leaf tissue, called photosynthetically active radiation (PAR) and the capacity of vegetation to accumulate carbon to increase biomass (Knapp et al. 2014). Hence, variations in NPP are the result of the PAR reaching the canopy, the amount that is absorbed (APAR), and conversion efficiency (Knapp et al. 2014). Annual NPP in MODIS (MOD17A3) is calculated by subtracting maintenance and growth respiration costs for leaves, fine roots, and woody tissue from daily gross primary productivity (GPP), adjusted for different biomes (Running and Zhao 2015). Because NPP is the net carbon gain by plants stored in plant biomass tissue we refer to NPP as biomass accumulation in plants. The allocation of NPP between the different parts of the plant is not equal; NPP can be allocated aboveground or belowground.

#### The capacity of ecosystems to supply ecosystem services

**Fig. 2:**
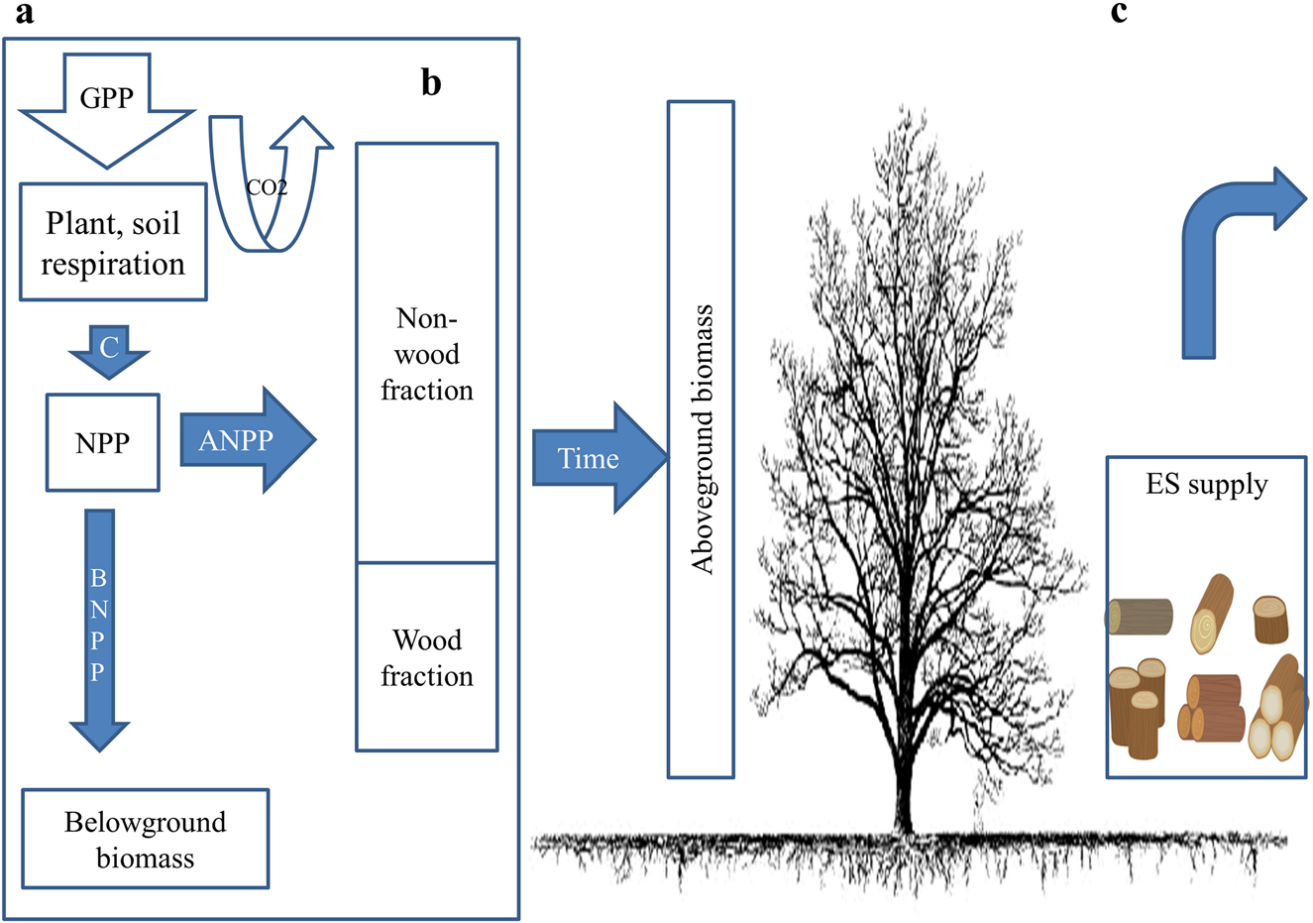
Schematic overview showing the capacity of ecosystems to supply ecosystem services, with timber as an example. **a** The gross primary productivity GPP is the source of the carbon available in forests ecosystems and NPP is the carbon available after plant and soil respiration which is allocated above (ANPP) and belowground (BNPP) as biomass, **b** the amount of aboveground biomass that is used to supply timber including wood and non-wood fractions, and **c** the accumulation of aboveground biomass of standing trees over time, and the supply of timber

The capacity of an ecosystem to supply an ecosystem service depends on the amount of aboveground biomass that is used to supply the ecosystem service (Fig. 2).

To assess the capacity of an ecosystem to supply ecosystem services FANNP, we used Eq. 1. The parameters of the equation were based on NPP allocation models from different studies that simulate the distribution of NPP in above and belowground, and the distribution of aboveground NPP in different parts of plant tissues (Table 1). All models were applied for each EA per BSU, per year.

1 $${\mathrm{FANPP}}x_{i,y} = \beta \times {\mathrm{ANPP}}x_{i,y}$$

2 $${\mathrm{ANPP}}x_{i,y}{\mathrm{ = }}\gamma \times {\mathrm{NPP}}x_{i,y}$$

**Table 1:** Linking ecosystem services, the capacity of ecosystems to supply biomass and the fraction of biomass used to supply ecosystem services

Ecosystem service	The capacity of ecosystems to supply ecosystem services		
	Aboveground biomass (*γ*)	Aboveground biomass that is used to supply ecosystem services (*β*)	References
Pastures for grazing cattle	For grassland and savannah the *γ* is 0.5	For grassland the *β* is 0.33	Sarmiento and Pinillos (2001)
	For woody savannah the *γ* is 0.6	For savannah the *β* is 0.30	Hui and Jackson (2006)
	For natural mixed the *γ* is 0.7	For woody savannah the *β* is 0.25	Scurlock et al. (2002)
		For natural mixed the *β* is 0.10	
Timber	For tropical forests the *γ* is 0.8	For tropical forests the *β* is 0.20	Aragão et al. (2009)
			Malhi et al. (2011)
Oil palm FFB	*F*or oil palm the *γ* is 0.96	For oil palm FFB the *β* is 0.45	Corley and Tinker (2008)
			Kotowska et al. (2015)
Carbon sequestration	All biomass is relevant for carbon sequestration	$x{\mathrm{NEP}}_{i,y} = x{\mathrm{NPP}}_{i,y} - x{\mathrm{Hr}}_{i,y}$ ^a^	Ott et al. (2015)

^a^To determine the capacity of ecosystems to sequester carbon and ecosystem services supply we use net ecosystem production. Net ecosystem production (NEP) is defined as the net carbon gain after plant and heterotrophs respiration but excluding disturbances (e.g. fire) which are not common for the study area. *x*NEP_*i,y*_ is net ecosystem production at each EA(*x*) (forest, grassland, savannah, woody savannah, natural mixed and oil palm), at year *y*. *x*NPP_*i,y*_ is NPP from MODIS MOD17A3 at given ET *x* per BSU(*i*) in year(*y*)

In Eq. 1, *β* is the part of the aboveground biomass that is used to supply each ecosystem service derived from literature (see Table 1), and ANPP*x*_*i,y*_ is the annual supply of aboveground biomass at given ecosystem *x*, at location *i*, at year *y*. In Eq. 2, *γ* is the amount of aboveground biomass derived from literature (see Table 1), and NPP*x*_*i,y*_ is MOD17A3 NPP at given ecosystem *x*, at location *i*, at year *y*. For example, the capacity of grassland ecosystem to supply pastures for grazing cattle in year 2014 at given BSU (with size of 21.4 ha) is the amount of aboveground biomass for grasslands (the NPP derived from MOD17A3 multiplied by 0.5), multiplied by 0.33, to specify the part of the aboveground biomass that is used to supply pastures for grazing. For each EA, we calculated the arithmetic mean and the standard deviation of the capacity to supply ecosystem services, and we used time series and box plots to show annual fluctuations for 14 years between 2001 to 2014.

#### Comparing ecosystem capacity and ecosystem services supply

To understand extraction–regeneration patters, we compared the capacity of each EA to supply ecosystem services with the supply of ecosystem services between 2010 to 2014. While for regulating services (e.g. carbon sequestration), the capacity equals the supply of ecosystem services, for provisioning services, where biomass is extracted, the supply of ecosystem services can be lower, equal or higher than their capacity to supply biomass (Hein et al. 2015; 2016). To assess the balance between extraction of ecosystem services and regeneration of ecosystems, we estimated the supply of ecosystem services based on equations and national statistics, and we compared this value with the capacity of each ecosystem to supply ecosystem services.

To estimate the supply of timber, we used Eq. 3. We split forest ecosystems in upland and lowland forests because tree species harvested at mountain ecosystems are different than those harvested at low altitude. We used 1500 m above sea level as an altitude threshold to split upland and lowland forest, however, we recognize that tree species composition at this altitude is heterogeneous and this threshold simplifies the reality. In Eq. 3, St is timber supply in ecosystem *f* (upland or lowland) expressed in tons of timber at given year *y*. In Eq. 3, *h* is the amount of timber harvested (in m^3^) per year *y*, multiplied by the average standing trees biomass (243 ton) of commonly harvested tree species in the study area, and the average wood density for tropical forest (0.6 ton/m^3^) (FAO 2010; Ideam 2011a; Oliver 2013; Phillips et al. 2011).

3 $${\mathrm{St}}_{y,f} = h_y \times 243 \times 0.6$$

To estimate the supply of pastures for grazing cattle, we used Eq. 4. In Eq. 4, Sp is the supply of pastures to graze cattle at given grazing EA *x* at year *y*. Grazing ecosystems *x* are grassland, savannah, woody savannah and natural mixed ecosystem. In Eq. 4, *φ* is the annual cattle intake as estimated by Gaviria-Uribe et al. (2015), and *c* is the annual cattle stock per EA*x* based on statistics (Fedegan 2014) (see Supplementary Material).

4 $${\mathrm{Sp}}_{y,x} = \varphi \times c_x$$

To estimate the supply of oil palm FFB, we used Eq. 5. In Eq. 5, So is the oil palm FFB supply, *a* is the annual FFB harvest multiplied by 0.56, which is the dry matter content in FFB (Contreras et al. 2012; Fedepalma 2013; 2015) (see Supplementary Materials for more details on the parameterization of the equations).

5 $${\mathrm{So}} = 0.56 \times a$$

For carbon sequestration, although capacity and supply are considered to be equal, removing biomass by harvesting timber, oil palm FFB and grazing pastures decrease the stock of carbon. We compared the estimated supply of timber, oil palm FFB and pastures grazed by cattle (from Eqs. 3, 4 and 5), with the capacity to sequester carbon for each EA.

## Results

### Ecosystems Supply of Aboveground Biomass

**Fig. 3:**
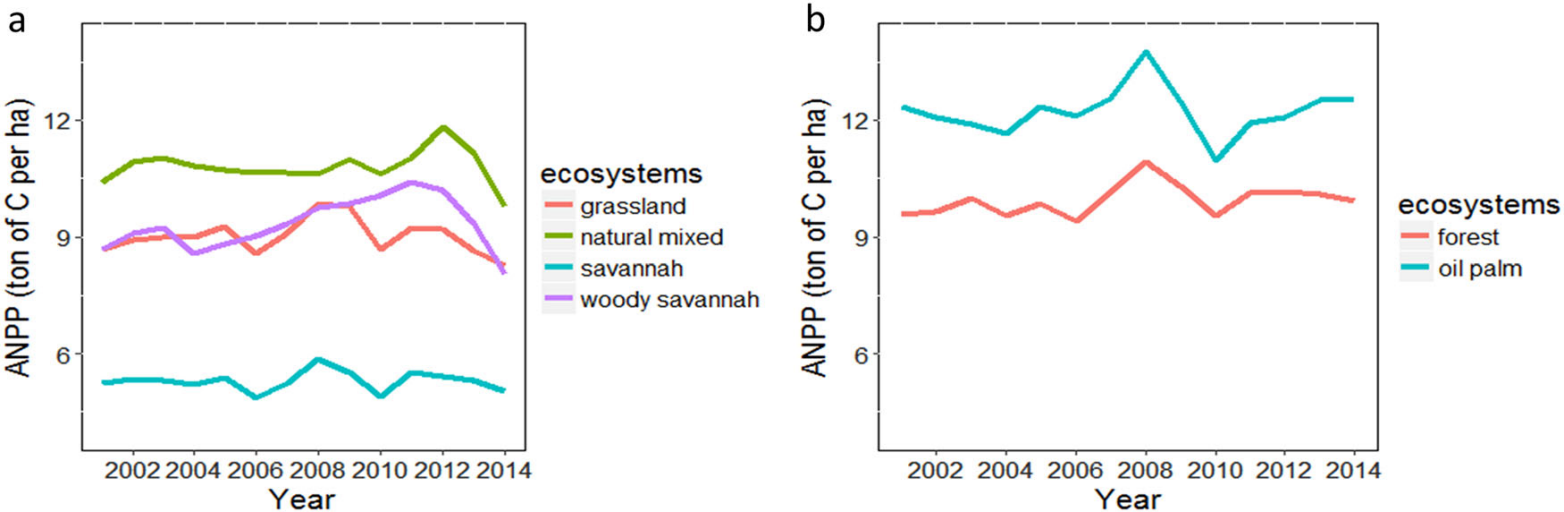
Time series showing annual fluctuations in the supply of aboveground biomass for each ecosystem. In **a** the ANPP for grazing ecosystems; grassland, woody savannah, savannah and natural mixed ecosystem, and in **b** ANPP for forest and oil palm plantations

For each ecosystem, fluctuations in the supply of aboveground biomass were assessed using the ANPP (Fig. 3). The difference between the lowest ANPP in the year 2014 and the highest value in 2008 for grassland ecosystems was 1.9 ton of carbon per hectare on average. When looking at differences per hectare between grazing ecosystems we observed that the highest ANPP was in the natural mixed ecosystem and the lowest in savannahs (Fig. 3a). For forests ecosystem, the annual ANPP increased 9.9 ton of carbon per hectare over the period from 2001 to 2014. Moreover, the annual ANPP in forest ecosystem fluctuated from 9.4 (±2.6) ton of carbon per hectare in 2006 to 11 (±2.6) ton of carbon per hectare in 2008 (Fig. 3b).The annual ANPP in oil palm was higher in the year 2008 compared to 2010 (Fig. 3b). Fluctuations in the ANPP reflect the sensitivity of NPP to changes in climatic conditions (e.g. rainfall pattern, light and water availability). Moreover, changes in the ANPP can be related to climate phenomena such El Niño and La Niña, which were particularly strong in 2008 and 2010, respectively (Ideam 2011b). These climatic phenomena increase water stress conditions stimulating the allocation of NPP to increase root system biomass in deep soil layers to overcome water shortages. The increase of root system biomass can decrease the availability of energy and carbon in other tissues such as leaves and diminishing photosynthetic activity.

### Ecosystems Capacity to Sequester Carbon, to Supply Pastures for Grazing Cattle, Timber and Oil Palm FFB

#### Carbon sequestration

**Fig. 4:**
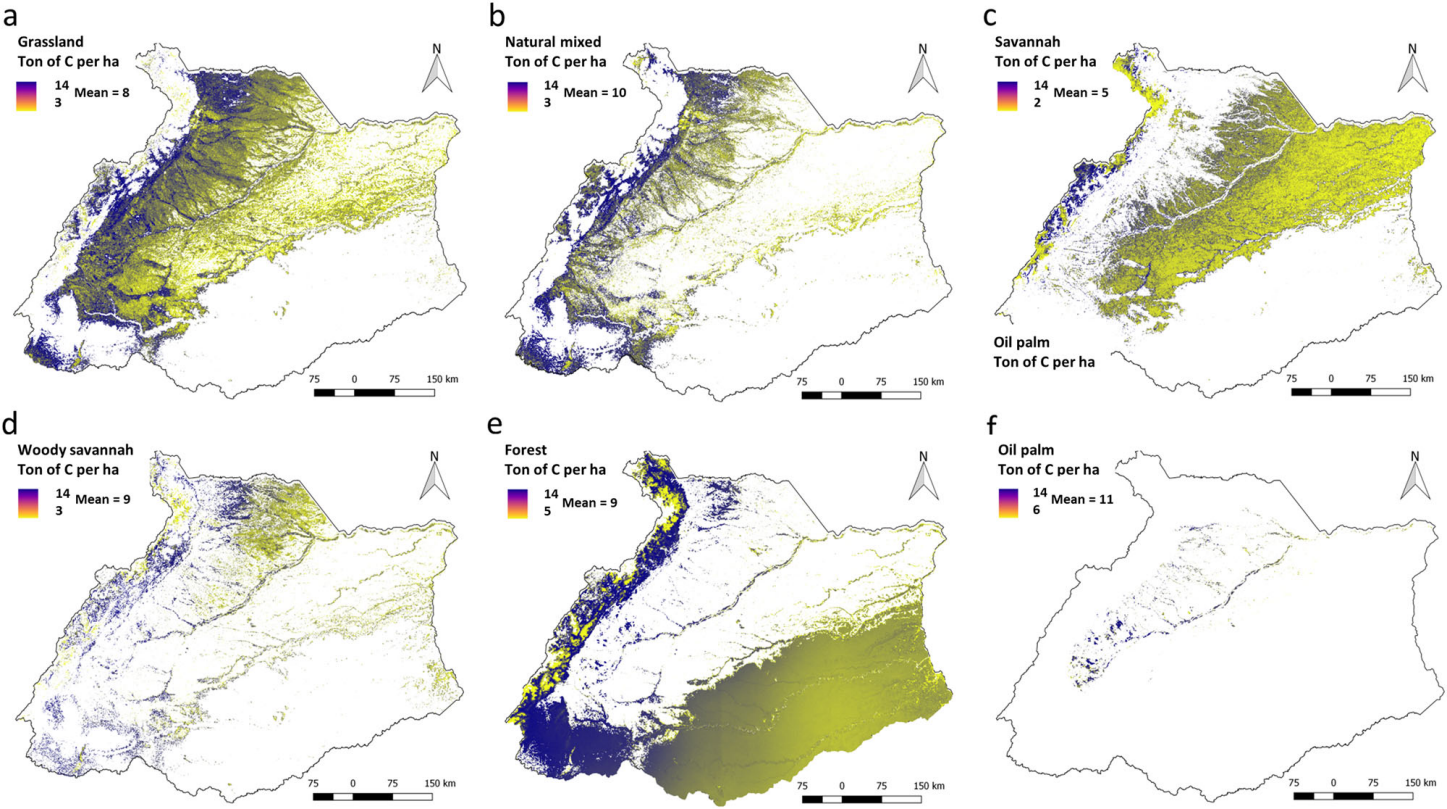
The average capacity to sequester carbon (NEP) in **a** forest ecosystem, **b** grassland ecosystem and **c** natural mixed ecosystem, **d** woody savanna, **e** forest and **f** oil palm, for the period 2001–2014

There is a clear variation in the capacity of ecosystems to sequester carbon between the ecosystems, defined by the land cover types. With an annual mean of 11 ton of carbon per hectare oil palm plantations had the highest capacity (compared to other ecosystems) to sequester carbon for the period 2001 to 2014. However, carbon is released into the atmosphere at the end of the harvesting cycle of the plantation (usually after 25 to 30 years) when oil palms are cut and usually replanted. The biomass from felled oil palm trees will be mostly released into the atmosphere through the burning of felling residues to clear the fields for planting. The capacity of the forest to sequester carbon was 9 ton of carbon per hectare on average. This value was lower than the capacity to sequester carbon in the natural mixed ecosystem, equal to woody savannah and higher than grassland (8 ton of carbon per hectare) and savannah (Fig. 4).

**Fig. 5:**
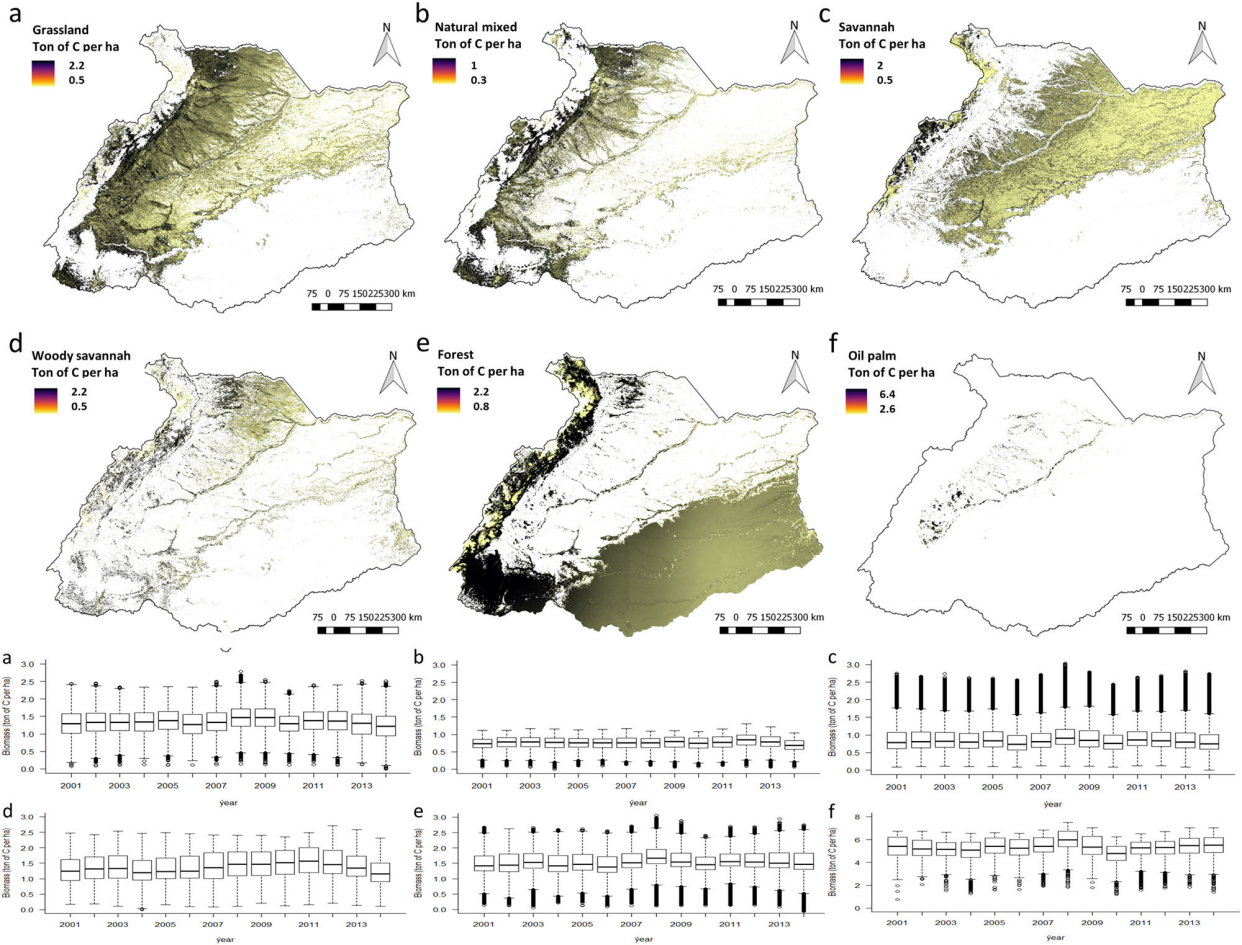
Maps showing the capacity to supply ecosystem services in **a** grassland, **b** natural mixed, **c** savannah, **d** woody savannah, **e** forest and **f** oil palm. Box plots showing capacity fluctuations between 2001 and 2014 for the same ecosystems

Adding more information about the altitude and vegetation density provides insight about the spatial patterns in the capacity of ecosystems to sequester carbon. In Fig. 1, we mapped the altitude and the vegetation density, and in Fig. 4 the spatial variation of the capacity to sequester carbon in grasslands, natural mixed, savannahs, woody savannahs, forests and oil palm ecosystems. The capacity to sequester carbon was high in the southwest of the river basin and in the eastern slopes of the Andes (Fig. 4), overlapping with locations where the density of trees is high (Fig. 1b). Conversely, the capacity to sequester carbon was low in the north-east plains of savannah and grassland ecosystems (Fig. 4a, c), overlapping with locations where the density of trees is low and with locations at high altitude (Fig. 1). Moreover, the capacity to supply pastures for grazing cattle, timber and oil palm FFB, followed the same spatial pattern as described for carbon sequestration (Fig. 5).

#### Pastures for grazing cattle

The capacity to supply pastures for grazing cattle did not largely fluctuate over time among grazing ecosystems (Fig. 5). Moreover, there were similarities between grasslands and woody savannah ecosystems, and between natural mixed and savannah ecosystems (Table 2). However, the NPP in woody savannah was higher compared to grassland, where most of the NPP was aboveground (Table 2). Because the non-grazed fractions (e.g. woody vegetation, trees) were higher in woody savannah ecosystem compared to grassland, the estimated biomass available to be grazed by cattle resulted in similar values for both ecosystems (Table 2). The capacity to supply pastures for grazing was similar for grasslands and woody savannah despite the NPP and the ANPP was higher for woody savannahs compared to grassland (Table 2). Likewise, the capacity to supply pastures for grazing was similar for natural mixed and savannah, despite that the NPP and the ANPP in natural mixed was more than twice the savannah ecosystem (Table 2).

**Table 2:** Net primary productivity, aboveground biomass and capacity to supply pastures for grazing cattle in ton of C per ha (mean and SD for the period 2001–2014)

Ecosystem	Primary productivity and aboveground biomass		Capacity
	NPP	ANPP	FANPP
Natural mixed	10.8 ± 2.6	7.6 ± 1.8	0.8 ± 0.2
Grassland	9.0 ± 2.5	4.5 ± 1.3	1.4 ± 0.4
Savannah	5.3 ± 1.9	2.6 ± 1.0	0.9 ± 0.03
Woody savannah	9.3 ± 3.0	5.6 ± 1.8	1.4 ± 0.05

We used a higher *γ* for natural mixed than for savannah ecosystem (0.7 vs 0.5 tons of carbon per ton of NPP), reflecting more trees in natural mixed compared to savannah ecosystem. However, in the natural mixed ecosystem, the capacity to supply pastures for grazing was lower compared to grassland and savannah (Fig. 5), because a large part of the biomass in the ecosystem consists of trees not grazed by cattle. The combined effects of both aspects are shown in Fig. 5. The higher NPP in non-grazed fractions such as trees also indicates that the carbon stock is higher in natural mixed ecosystem compared to savannah and grassland (Table 2).

#### Timber

The forest ecosystem capacity to supply timber showed substantial spatial variation. Whereas in forests located on high altitudes, the capacity to supply timber was below 0.8 ton of carbon per hectare, in forests located in the southwest corner of the river basin this capacity was generally above 2.2 ton per hectare (Fig. 5). Moreover, in a large proportion of the forest ecosystem the average capacity to supply timber was 1.2 ton per hectare between 2010 and 2014, however, the annual capacity to supply timber was 1.5 ton of carbon per hectare in 2014 (Fig. 5). Differences in ecosystem conditions (e.g. age, species, density, rainfall, altitude) influence forests photosynthetic activity, NPP allocation, and thereby the annual increment of harvestable timber. The spatial variation of these fraction followed different patterns related to differences in conditions such as altitude and tree density (Fig. 1).

#### Oil Palm FFB

Oil palm plantations showed a non-homogeneous distribution of the capacity to supply FFB over time (Fig. 5). On average, the capacity to supply FFB was 5 ton of carbon per hectare between 2001 and 2014, however, maximum values (7 ton of carbon per hectare) were observed in 2008 and minimum values (2 ton of carbon per hectare) in 2010 (Fig. 5f). Differences in the capacity to supply FFB between years can be influenced by differences in the condition (e.g. rainfall conditions, age, disease occurrence) of the plantation.

### Comparing ecosystems’ capacities to ecosystem services supply

#### Carbon sequestration

In the case of carbon sequestration, the capacity to supply the service equals the supply of the service because in the conceptualisation of the SEEA-EEA supply of regulating services does not involve an extraction or active use of the ecosystem and is therefore, in principle, always sustainable (Hein et al. 2016). Nevertheless, differences in the amount of biomass removed by harvesting influence the amount of carbon stored in each ecosystem over time. The amount of biomass removed from oil palm plantations was a bit higher (56%) compared with natural mixed (41%) and grassland (40%), higher compared with savannah (22%) and lowland forest (10%), and very high compared with woody savannah (6%) and upland forest (1%) (Table 3). Biomass in standing oil palms has a 25–30 years life span in which a substantial part of the NPP allocated aboveground is removed as FFB, and only a small part of the energy and carbon allocated belowground will remain for long periods of time. If carbon sequestration constitutes an avoided flow of carbon to the atmosphere and time is considered, then oil palm plantations will be the ecosystem with the lowest capacity. Forests, grassland and savannah ecosystem have a similar capacity to sequester carbon (in ton per hectare per year), however forests and woody savannah will provide more benefits as more carbon will be kept in the ecosystem for longer periods of time. Natural mixed, grassland and savannah ecosystem are intermediate as the biomass exported by these ecosystems depends on the amount of cattle raised by each ecosystem, the number of trees and the amount of vegetation not eaten by herbivorous.

**Table 3:** Comparing average annual capacity of ecosystems to supply timber, oil palm FFB and pastures with the annual ecosystem services use in seven ecosystems

Ecosystem		Ecosystem service		Capacity		Ecosystem services use	
	Area (in ha)	Service	Unit	Ton/year	Ton/ha/year	Ton/year	Ton/ha/year^a^
Forest							
Upland	2,124,561	Timber harvesting	Ton of timber	6,232,000	2.93	58,000	0.03
Lowland	13,050,873			54,140,000	4.15	5,492,000	0.43
Natural mixed	2,319,961	Pasture grazing	Ton of pasture in dry matter	5,598,000	2.41	2,289,000	1.01
Grassland	6,792,129			26,468,000	3.90	10,644,000	1.60
Savannah	8,654,258			19,842,000	2.29	4,422,000	0.52
Woody Savannah	835,576			13,234,000	15.84	829,000	1.01
Oil palm	107,154	FFB harvesting	Ton FFB in dry matter	1,704,000	15.90	954,000	9.07

^a^Deforestation takes place on 391 ha in upland and on 37,691 ha in lowlands forest each year (annual average between 2010 and 2014), however, to compare capacity with supply it was calculated from the area covered by each ecosystem in ton/ha/year

#### Pastures for grazing cattle

Grassland and savannah ecosystem together provided 71% of the total capacity to graze cattle provided by all grazing ecosystems (Table 3), supporting 4.4 million cattle heads in 15 million hectares (for details on cattle stocks see Supplementary Material). However, grazing cattle removed 40% of the grassland capacity to supply pastures and 22% of the savannah (Table 3). Woody savannah provided 20% of the total capacity to graze cattle, supporting 243 thousand heads in 819 thousand hectares. Natural mixed systems provided 9% of the total capacity to graze cattle, supporting 660 thousand cattle heads in 2 million hectares (Table 3). However, grazing cattle removed 41% of the natural mixed capacity to supply pastures and 6% of the woody savannah. A substantial portion of the capacity to supply pastures is removed by grazing cattle in grassland (40%) and natural mixed (41%) ecosystems, because conditions (e.g. water availability, soil type, palatability) and management (e.g. infrastructure) in these two ecosystems are more favourable to graze cattle. In woody savannah most of its capacity is not removed by grazing cattle probably because the conditions in this ecosystem are less suitable for grazing (e.g. poor fertile soils, low nutritional quality grass species), and management is difficult (e.g. remote areas, flooded during raining season).

#### Timber

Whereas the capacity to supply timber was around 100 times the annual timber supplied in upland forests, this capacity was 10 times higher than the timber supplied in lowland forests (Table 3). Moreover, the forests (up and lowlands) capacity to supply timber was 10 times higher than the timber harvested. Yet, forest ecosystem covered 15 million hectares by 2014, but annually 38,000 hectares were deforested (Ideam 2015). Accordingly, it can be considered that the annual capacity to supply timber was enough to cover the annual supply of timber. However, two additional location-specific issues need to be taken into account. First, most of the annual capacity to supply timber can be available but not suitable for harvest. Forests inside national parks (e.g. La Macarena, Tuparro) and indigenous reserves have a high capacity to supply timber, however, timber harvesting is forbidden by law in national parks and is controlled inside indigenous reserves. The area under national parks covers 1.5 million hectares, and inside indigenous reserves 8 million hectares (Correa et al. 2005). Moreover, this biomass can be inaccessible for timber harvesting, for example, in forests located in pronounced slopes, flooded tropical rainforests and remote forests with no access roads. Second, timber harvesting takes place in specific deforestation areas considered as hotspots, mostly in lowlands forests located in the southwest portion of the Orinoco river basin (Etter et al. 2006). The capacity to supply timber may not be able to compensate the supply of timber in hotspot deforestation areas over long periods of time, putting the sustainability of these hotspot areas at risk. Our analysis shows that changes in land cover and land-use alter the future capacity of ecosystems to supply ecosystem services both in terms of types of ecosystem services and in a number of services that can be sustainably generated.

#### Oil palm FFB

Adult oil palms mobilize a large portion of their annual additions of carbon (>80% NPP) to produce FFB biomass. Although adult oil palms have a high capacity to supply FFB biomass (16 ton of carbon per hectare per year), the harvesting of FFB removes 56% of this capacity (9 ton of carbon per hectare per year). However, in non-producing young oil palms (younger than 34 months) most of the biomass remains in the system as there is no biomass removal by FFB harvesting (not measured in this study). High rainfall leading to floods, long periods of drought, poor nutrient availability and diseases decrease oil palm photosynthetic activity and lead to lower allocation of NPP to fruit tissues, which reduces FFB supply.

#### Understanding the link between capacity and supply

In order to test the applicability of information on ecosystem capacity derived from remote sensing, a comparison has been made between capacity and flow of ecosystem services. In principle, provided the estimates are of sufficient accuracy, flows exceeding capacity indicate unsustainable use. Such an assessment cannot be made by comparing average values over ecosystem types but the comparison needs to be for individual BSU (on a pixel by pixel basis). We are currently testing the overall approach and cannot ensure sufficient accuracy on a BSU by BSU level. We therefore only compare spatial averages of flow and capacity, in order to get a first idea of the order of magnitude of the overall difference (Table 3). Hence, the information in Table 3 may conceal that overharvesting of ecosystem services is taking place at specific locations. For example, not all timber is available for logging such as timber in protected areas hence ideally harvest patterns and capacity should be compared at the level of the BSU (see also Schröter et al. 2014). Yet, overall, it appears as if timber harvesting rates are well below the average annual increment of harvestable timber. At the other hand, it seems as if a significant portion (1.6 ton out of 4 ton) of grass in pastures is grazed by livestock. Note that in this case a 100% use rate of the capacity is not likely for instance because some of the palatable biomass is produced during periods of high supply (wet season) or in areas not accessible to cattle. In the case of oil palm, it is likely that most of the produced FFB biomass is harvested, although there may be losses of FFB due to for example diseases. In this case, the difference between crop and flow, therefore, may reflect inaccuracies in our capacity estimates, inaccuracies in harvest statistics as well as crop losses, or a combination thereof.

## Discussion

### Using Remote Sensed Information in Ecosystem Accounting

Ecosystem accounting is an integrated framework developed to incorporate measures of ecosystems and ecosystem services into the structure of national accounts (Hein et al. 2015; Obst and Vardon 2014; United Nations et al. 2014b). Ecosystem accounting is spatially explicit approach and includes an assessment of the capacity of ecosystems to supply ecosystem services (United Nations et al. 2014b). However, such assessment requires spatial explicit information which is not always available. Accordingly, the SEEA-EEA noted that data scarcity especially at national and subnational levels is one of the main sources of uncertainty for the physical measurement of the capacity of ecosystems to supply ecosystem services (United Nations et al. 2014b). Our study explored the feasibility of compiling remote sensed spatially explicit information following ecosystem accounting guidelines to assess the capacity of ecosystems to supply ecosystem services at the level of a river basin. Our results show that remote sensed information can be used to determine accounting units following the ecosystem accounting guidelines. Ecosystem accounting needs land cover information as the starting point to determine the spatial distribution of ecosystems using spatial explicit accounting units (United Nations et al. 2014b). However, land cover maps at the national level are not regularly produced every year, hindering the assessment of annual changes in the spatial distribution of ecosystems by monitoring accounting units such as EA. The MODIS MCD12Q1 land cover product is annually classified based on training and test sites providing global cross-validated information with 76% overall accuracy among land cover classes (Friedl et al. 2010). We believe that this land cover product can be used to support the determination of ecosystem accounting units. Moreover, this product can be used as the starting point for the assessment of changes in ecosystems and ecosystem services. Other sources of spatially explicit information such as cadastral data, elevation, soil type and land cover maps, and aerial photography can be used to complement the MODIS land cover product to determine spatial units. Our results also show that remote sensed information can potentially be used to assess the capacity of ecosystems to supply ecosystem services following the ecosystem accounting guidelines. NPP is highly sensitive to changes in conditions such as rainfall pattern, water and nutrients availability (Knapp et al. 2014), making NPP a suitable indicator to assess the capacity of ecosystems to supply ecosystem services. The approach applied in this study was based on the dependence of biomass harvest on plant NPP and on NPP allocation. We found that each ecosystem has a different capacity to supply ecosystem services that varies in space and time. NPP can be linked with the supply of multiple ecosystem services, making the comparison of ecosystem services supply and capacity possible. The MODIS NPP product combines information from land cover, meteorology and vegetation index products, with their own uncertainty that can propagate and influence MODIS NPP information (Zhao et al. 2010). Uncertainties and validation of these products have been regularly assessed (Turner et al. 2006; Zhao et al. 2006), and improvements on the MODIS NPP algorithm have been released (Running and Zhao 2015). However, NPP has a limited use for assessing ecosystem services not direct related with primary productivity such as hydrological and cultural services. Remote sensing products such as MODIS global evapotranspiration product MOD16, METEOSAT-8, NOAA and Tropical Rainfall Measuring Mission (TRMM) can be used to support the assessment of hydrological ecosystem services (Carvalho-Santos et al. 2013). New missions such as SENTINEL, SAR and LiDAR sensors, and initiatives such as the Group on Earth Observations Biodiversity Observation Network (GEOBON) offer new opportunities to support the assessment of ecosystems and their services and to remotely monitor change in ecosystems, ecosystem services and biodiversity (Tallis et al. 2012).

### Can Capacity–Supply Models be Used to Analyze Sustainability?

Ecosystem accounting focuses on the assessment of ecosystems and their services providing integrated information required to assess environmental sustainability (United Nations et al. 2014b). The supply of ecosystem services involves the extraction and harvest of resources. Harvest and regrowth rates in ecosystems determine the sustainability of ecosystem use (United Nations et al. 2014b). A first step towards the analysis of sustainability in ecosystems has been the use of spatial models that integrate the capacity of ecosystems to supply services and the supply of ecosystem services (Burkhard et al. 2012; Schröter et al. 2014; Villamagna et al. 2013). These models showed that the harvest of ecosystem services can exceed the capacity of ecosystems to supply the service putting ecosystems sustainability at risk. However, the assessment of the capacity of ecosystems to supply ecosystem services is challenging because of the ecosystem’s extent and condition (e.g. water, nutrients, temperature, rainfall) change in time and space. We use NPP to estimate the capacity of ecosystems to supply ecosystem services and compare this with average use of ecosystem services (note that in the conceptual framework of the SEEA-EEA, the use of an ecosystem service equals, by definition, the supply of the ecosystem service). For provisioning ecosystem services, the supply can be lower, equal or higher than the capacity of the ecosystem to supply ecosystem services. The spatial variation is an important aspect to consider in the assessment of the sustainability of ecosystems at large scale. We showed that the forest capacity to supply biomass at the scale of the whole river basin exceeds the amount of timber biomass harvested. Timber harvests take place in dedicated forest patches, where in the year of harvest, extraction exceeds regrowth. Our study indicates that at the level of the basin there is no overharvesting of timber. However, not all forests are subject to timber harvesting, for example, because they are protected or inaccessible. In the future, our analysis can be refined by comparing regrowth and extraction rates in areas that are harvested (see e.g. Schröter et al. 2014). The harvested area can be derived from forest concessions (as well as from remote sensing images if the annual imagery of sufficient resolution ≤30 m for a time period of at least one logging cycle is available). An additional future refinement is that the assessment of the overall sustainability of ecosystems by capacity–supply mapping models should consider the inter-annual variation of the capacity of ecosystems to supply ecosystem services. As we show, there can be substantial variation in biomass regrowth between years, for instance, due to different weather patterns. Annual budgets simplify the capacity of ecosystems–supply dynamics, climate events such as droughts during El Niño influence ecosystem services supply by altering the capacity of ecosystems to supply ecosystem services at specific locations. Such refinements can enhance the accuracy and thereby the applicability of our approach. Potentially, this could lead to an efficient way of measuring the sustainability of ecosystem use by comparing local patterns in regrowth and extraction rates. Where this measurement system can be embedded in the SEEA Ecosystem accounts, extraction rates for provisioning services can be linked to effects on regulating and cultural services (see Hein et al. (2016) for a potential way forward on linking ecosystem use to capacity for different types of services).

### Implications for SEEA-EEA

The SEEA-EEA considers the spatial assessment of the capacity of ecosystems to supply multiple ecosystem services as central to understand how human activities change ecosystems and how these changes are related to the future generation of ecosystem services. Specifically, this concept helps to define ecosystem use patterns, to develop and evaluate alternative use scenarios, and to assess ecosystem degradation (Edens and Hein 2013; United Nations et al. 2014b). However, the assessment of the capacity of ecosystems to supply services is challenging because ecosystems are complex dynamic systems influenced by many factors (e.g. changes in soil pH, water availability, climate, light). Changes in land use for instance, by switching from forest to agriculture modify ecosystem conditions (e.g. by polluting downstream waters), and the capacity of ecosystems to supply fresh water, compromising the supply of fresh water in the future. Ecosystem capacity indicators should be able to spatially reflect changes in ecosystem condition in space and time, and the implications in the future ecosystem services supply. In our study, we explored if NPP can be used as an indicator for the assessment of the capacity of ecosystems to supply ecosystem services (timber and FFB harvesting, carbon sequestration and pastures for grazing cattle).

Our study included MODIS NPP to assess capacity by assessing the amount of aboveground biomass that is used to supply an ecosystem service. Whereas assessing the supply of aboveground biomass give us insights about ecosystem regeneration patterns, NPP allocation is key to link the supply of aboveground biomass with a specific ecosystem service. However, the assessment of NPP allocation is challenging as ecosystems are dynamic systems where the amount of aboveground biomass allocated to supply an ecosystem services can change in time and space. We used different NPP allocation models to assess the amount of aboveground biomass that is used to supply ecosystem services. However, the information provided by these models was not specific for the Orinoco river basin, it was adjusted from different countries such as Malaysia for oil palm, and China, Uruguay, and Venezuela for grazing pastures. Ecosystem conditions are clearly different in the locations where these models were developed increasing uncertainty to our results. MODIS NPP is a powerful tool to assess the spatial variation of the capacity of ecosystems to supply ecosystem services in large areas, however, can be limited in contexts where a high level of detail is required such as municipality and local level, e.g. in Remme et al. (2015) and Villamagna et al. (2013). However, the moderate spatial resolution of this sensor can be compensated with the dimensions of its swath that covers 2.3 by 10 km per scene every day, the temporal resolution where products are available every 8–16 days, month and year processed from level 2 up to level 4 where products are modelled and produced at high quality. New developments in remote sensing can have an important role in the further development of SEEA-EEA ecosystem accounting by providing information to assess harvesting-regeneration patterns, monitor ecosystem change, and to assess the future generation of ecosystem services. New opportunities by combining remote sensing with economic and social information can be useful for the assessment of current and future ecosystems use, alternatives scenarios, and ecosystems degradation towards sustainable use of ecosystems.

## Conclusion

There is a growing interest in ecosystem accounting to support the protection of ecosystems and the future supply of ecosystem services. Ecosystem accounting was developed as an experimental system towards the integration of environment and economic information into the system of national accounts. Ecosystem accounting includes the assessment of ecosystems’ capacity to supply multiple ecosystem services (United Nations et al. 2014b). Our study provided insights on (i) how the capacity of ecosystems to supply ecosystem services can be assessed and (ii) how remote sensing can be used to support this assessment in large areas. In our study, we proposed NPP as a suitable indicator to assess the capacity of ecosystems to supply timber, pastures for grazing cattle, oil palm FFB and to sequester carbon because NPP is sensitive to changes in ecosystem condition and changes in primary productivity that affect the supply of ecosystem services. However, more research on NPP allocation is required to improve current knowledge, on mapping the capacity of ecosystems to supply ecosystem services and ecosystem services supply. Annual land cover information from MODIS MCD12Q1 can be a potential source of information to assess land cover changes in line with the annual periodicity of ecosystem accounting, in particular for large, relatively homogeneous ecosystems as found in the Orinoco river basin. Our study explored MODIS primary productivity to provide spatial information for the assessment of the capacity of ecosystems. We found that MODIS NPP can be a powerful source of spatial information to assess the capacity of ecosystems at river basin scale such as the Colombian Orinoco. However, NPP is most relevant for provisioning and selected regulating services, much less so for cultural services. New developments in earth observation (higher spatial temporal resolution, new sensors) will complement currently available datasets for ecosystem assessments and for the integration of environment and economic information. The presented approach used for the assessment of the capacity of ecosystems to supply ecosystem services is a basis for further refinements that will allow developing capacity–supply models for ecosystem accounting and other applications.

## Electronic supplementary material

Supplementary Information
