# Supplementary material for: Assessing the Capacity of Ecosystems to Supply Ecosystem Services Using Remote Sensing and An Ecosystem Accounting Approach
Source: Environ Manage. 2018 Sep 28;63(1):1–15. doi: 10.1007/s00267-018-1110-x (PMC6353808; doi:10.1007/s00267-018-1110-x)
Supplement: Supplementary file 1 — Supplementary Information [file 267_2018_1110_MOESM1_ESM.docx]

Appendix A. Supplementary material

**1. Data analysis tools**

We downloaded two images from MOD44B Vegetation Continuous Fields (VCF) product, two images from digital elevation model Global Multiresolution Terrain Elevation Data (GMTED2010), 56 images from MODIS MOD17A3 and 28 images from MODIS MCD12Q1. These images were downloaded from the online data pool, courtesy of NASA Land Processes Distributed Active Archive Center (LP DAAC), USGS/Earth Resources Observation and Science (EROS) Centre, Sioux Falls, South Dakota, <https://lpdaac.usgs.gov/data_access/data_pool>, to support land cover assessment. We downloaded 144 images containing global monthly heterotrophs respiration from the National Aeronautics and space Administration (NASA) Carbon Monitoring System (Collatz and Kawa, 2014; Ott et al., 2015). We used MODIS re-projection tool (MRT tool) to mosaic and re-project all MODIS images to UTM 19N WGS84 by nearest neighbour (Courtesy of NASA/LP DAAC). We used R studio packages, raster, sp, and ncdf, to resample, crop, stack, and subset all remote sensing images (Team RStudio, 2015). One map from Instituto de Investigación de Recursos Naturales Alexander von Humboldt Colombia (IAvH) was used to mask and clip all remote sensing images to match the extent of the Orinoco River Basin.

We create one raster stack by stacking two raster stacks; one containing 14 images from MOD17A3 yearly NPP and one containing 14 images MCD12Q1 land cover types, (yearly stacks from 2001 until 2014). We subset 6 ecosystems that overlay thematic classes in MCD12Q1 stack with NPP values from MOD17A3 stack. We used this subsets to assess the capacity of ecosystems to supply biomass. In addition, to assess carbon sequestration we create 12 raster stacks containing annual mean heterotrophs respiration (Hr) values by stacking 144 monthly Hr images (12 per year) from year 2003 until 2014. We create one raster stack containing 12 Hr raster stacks. We used this raster stack to subtract Hr from the subset ecosystem maps containing NPP values. We used R studio software for the assessment of spatial and temporal variation of the capacity of ecosystems to supply ecosystem services (Team RStudio, 2015). We used histograms, level-plots, xy time series and basic statistics to analyse spatial and temporal variation of the capacity of ecosystems to supply ecosystem services by using R studio packages (raster, rasterVis, rts, lattice and ggplo2) (Team RStudio, 2015).

**2. Calculating ecosystems capacity to supply ecosystem services**

**Ecosystem services and ecosystem type**

Four ecosystem services relevant for the Orinoco river basin were selected; harvesting oil palm FFB, grazing pastures by cattle, harvesting timber, and carbon sequestration. The selection of these ecosystem services was based on their socio-economic and environment relevance. Oil palm plantations have been the main source of land use change in the river basin, increasing from 32 km^2^ in 1960 to 162 km^2^ in 2007 (Romero-Ruiz et al., 2012). Harvesting oil palm FFB is one of the most important economic activity in the river basin, as is the source of 16 employments per Km^2^ (Benavides, 2010; Romero-Ruiz et al., 2012). Grazing cattle is the human activity that covers most non-forested land, covering more than 5 million hectares (Fedegan, 2014). Forest ecosystem covers more than 60% of the river basin in which harvesting timber is one of the most important human activities (Lasso et al., 2010). Carbon sequestration is essential to provide ecosystem’s energy, carbon, and for climate change mitigation.

**Carbon sequestration**

Carbon sequestration can be defined as the annual rate of atmospheric CO_2_ added to carbon pools contributing in climate change mitigation (Lorenz, 2013; Naidoo et al., 2008). The capacity of ecosystems to sequester carbon can be determined by subtracting heterotrophs respiration (*Hr*) from NPP as the net carbon gain after plants respiration. The link between NPP, plant and heterotrophs respiration can be captured by Net Ecosystem Production which accounts for carbon fixation by plants through photosynthesis and carbon losses by autotrophs and heterotrophs respiration, excluding disturbances (e.g. fire) (Potter et al., 1999; Potter et al., 1993; Poulter et al., 2014). We used Net Ecosystem Production (*NEP)* to measure the capacity of ecosystems to sequester carbon between 2003 and 2014.

**Pastures for grazing cattle**

The capacity of ecosystems to supply pastures depends on ecosystem conditions (e.g. land-cover, grass species, soil type, altitude), and NPP allocation. In principle, pastures are a mix of different grass species (e.g. *Axonopus purpusii*, b*raquiaria humidicola*), shrubs (e.g. *Paspalum carinatum*), woody vegetation (e.g. *Curatela americana* ), trees and palms (e.g. *Mauritia flexuosa*)(Amézquita et al., 2013; Lasso et al., 2010). We used four different grazing ecosystems; grassland, savannah, woody savannah and natural mixed ecosystem. Grassland and savannah ecosystem were characterized by low tree land cover (less than 20%) but different characteristics (e.g. soil type, management, grass species, climate). Grassland were covered by improved grass species located in fertile soils in the mountains and well-drained plains close to the Andes. Savannah ecosystem is located in poor soils (acidic compact soils) with native grass species exposed to floods, burns and droughts in the east plains. Natural mixed and woody savannah ecosystem were largely covered by trees (more than 40%), however these two ecosystems had different characteristics. Natural mixed ecosystem were mainly located in the Andes mountains and foothills covered by large canopy trees mixed with agriculture systems in fertile soils, woody savannah were located in the east plains covered by woody vegetation and low canopy trees in riparian forests in poor fertile soils. In addition, the grazing capacity of ecosystems to supply pastures depends on NPP through ecosystem’s photosynthetic activity, NPP allocation between aboveground (ANPP) and belowground (BNPP) and the fraction allocated to increase pasture biomass. NPP allocation is determined by resources availability (e.g. nitrogen, phosphorous and water), climate (precipitation, temperature, evapotranspiration, sun light) and grazing behaviour (Gao et al., 2008; Gilmanov et al., 1997). Grazing stimulates BNPP allocation by increasing belowground biomass in roots, rhizomes and bulbs, however ANPP reflects the amount of biomass available for grazing and the fraction consumed by cattle (Grigera et al., 2007; López-Mársico et al., 2015). Hui and Jackson (2006) estimated that 40% to 90% of the total NPP is distributed belowground, however, in the tropics NPP allocation varies within the year, and ANPP can be close to 50% of NPP for grasslands and savannahs, 60% for woody savannahs and 70% in mixed ecosystems (trees and pastures (Sarmiento and Pinillos, 2001). Not all *ANPP* is available for grazing, NPP can be lost (e.g. consumed by other herbivorous, burned by fires, senescence, litter fall), or be not suitable for cattle (e.g. stand as biomass in trees, low palatability, inaccessible, flooded). The available fraction of ANPP can be close to 25–33% ANPP (Sarmiento and Pinillos, 2001). The fraction of biomass used for grazing in grassland is 33% , savannah 30%, woody savannah 25% and woody savannah 10% (Hui and Jackson, 2006; Scurlock et al., 2002).

**Timber**

Different conditions such as temperature, elevation, and precipitation influence forests distribution and structure, resulting in a wide variety of forests ecosystem in the Orinoco (e.g. tropical rainforest, tropical dry forests, tropical mountain systems)(Lasso et al., 2010; Pan et al., 2013). Forest capacity to supply timber for harvesting depends on tree NPP, the allocation of NPP above and belowground, and to different organs, such as woody tissue, flowers and fruits (Arag̃o et al., 2009; Malhi et al., 2011).We used forest productivity (NPP), NPP allocation and the woody fraction for the assessment of forests capacity to supply timber for harvesting. ANPP allocation in tropical forests can fluctuate from 64% to 84% NPP (Malhi et al., 2009). Wood fraction is the carbon flux into wood pools over time to increase timber biomass. We used information from prior studies that model the annual carbon added to wood fraction standing trees in tropical forests which averaged 40% of NPP (Malhi et al., 2011; Potter et al., 2013). We estimated that the wood fraction is 20% of aboveground biomass. We used a general approach assuming that wood fraction F*ANPP* did not differ between species, age, soil type, altitude and many other conditions, however we recognized that differences in condition change NPP allocation above and belowground, and to the wood fraction in tropical forests (Malhi et al., 2011).

**Oil palm FFB for harvesting**

The production of FFB biomass in oil palm (*Elaeis guineensis Jacq*.) depends on conditions such as age, water availability, temperature, fertilization and soil type (Corley and Tinker, 2008; Mejía, 2000). Age is an important factor to consider when assessing NPP allocation, NPP in young palms can be very low when they are transplanted from the nursery and very high in palms older than 8 years (Lamade and Bouillet, 2005; Tan et al., 2012). The production of FFB in Colombia reach an optimum level in oil palms older than 6 years, when the content of oils in the fruits increase (Narváez et al., 1996; Prada et al., 2012). In oil palms 8 to 15 years old, 96% total annual dry matter is stored in aboveground biomass, including trunk, fronds and brunches in palms (Corley and Tinker, 2008; Kotowska et al., 2015; Melling et al., 2008). We used MODIS 44B vegetation continuous fields (VCF) to map fully grown oil palm plantations, as we assume that a closed canopy is only present in oil palms plantations older than 8 years. We used a texture band and soil type to separate oil palm from forest in the land cover classification (Gutiérrez-Vélez and DeFries, 2013; Tan et al., 2013). Oil palm NPP is allocated aboveground to increase biomass in different parts of the palm such as canopy, stem fronds, and fruits. Melling et al. (2008) calculated that NPP allocation to FFB in oil palms older than 5 years was around 45% ANPP. For simplicity, we assumed that all oil palms in our study have closed canopy and were older than 8 years allocating 45% of their ANPP to the production of FFB. However we recognised that age, rainfall, soil type and other conditions influence the amount of NPP allocated to FFB production.

**3. Calculating ecosystem services supply**

**Carbon sequestration**

In principle, the amount of carbon sequestered by each ecosystem and added to terrestrial biomass can be seen as an avoided supply of carbon to the atmosphere (e.g. carbon stored in vegetation, microorganisms, animals)(Naidoo et al., 2008). Consequently, the capacity of ecosystems to sequester carbon and ecosystem services supply (as the avoided flow of CO_2_ to the atmosphere) are considered as equal (Schröter et al., 2014).

**Grazing ecosystems to supply pastures**

The supply of pastures grazed by cattle was deducted from annual cattle stock data, and the average annual pasture intake per head/year. The total annual average cattle stock between 2010 and 2014 was 5.2 million heads on average, obtained from the National federation of Cattle producers (Fedegan, 2014). To calculate the annual cattle stock per ecosystem, first, we overlapped the total annual cattle stock per department with the extent of each of the 4 grazing ecosystems (grassland, savannah, woody savannah and mixed ecosystem), assuming an equal distribution of cattle over the department. The average cattle stock between 2010 and 2014 per ecosystem was 4,4 million heads in savannah, 10,6 million in grassland, 2,2, million in natural mixed and 0,8 million in woody savannah (Fedegan, 2014). Second, to calculate the annual intake we used information from Gaviria-Uribe et al. (2015) and we assumed an average annual intake of 3.5 ton of dry matter /head/year (based on annual feed intake average of 9.6 kg of dry matter/day with 350Kg live weight. Finally, to calculate the supply of pastures grazed by cattle we multiplied the annual intake with the number of cattle per ecosystem.

**Timber harvesting**

Most of the timber harvested in the river basin comes from natural forest, mainly from tree species such as *Ocotea cyimbarum*, *Astronuim graveotens*, *Quercus humboldtii* and *Weinmannia tomentosa* (Ideam, 2011). Most of the timber harvested between 2010 to 2014 was for domestic consumption (only 1% of national timber harvested was exported) used for fuel and industrial wood (e.g. sawn wood, particleboard and pulp (FAO, 2010; Oliver, 2013). Timber was harvested legally and illegally, an estimated 56% of the total harvest was illegal (Muñoz and Carvajal, 2013). To account legal and illegal timber harvest, we used annual timber harvest data and we divided forest ecosystem according to their elevation in two broad categories; upland mountain tropical forests located above 1,500 meters and lowlands tropical forests below 1,500 meters. We obtained information about timber harvest from the program for deforestation monitoring and surveillance implemented by the National Institute for Hydrology, Meteorology and Environmental studies (IDEAM).

**Oil palm fresh fruit bunches (FFB)**

We gathered information from the national federation of oil palm producers concerning the annual FFB harvest in ton of FFB per hectare per year in the Orinoco River Basin, between years 2010 and 2014 (Fedepalma, 2015). We adjusted the fresh weight of fruit bunces to dry FFB weight to allow comparisons with oil palm capacity to supply oil palm FFB which is in ton of biomass. According to Contreras et al. (2012) the dry weight of oil palm *E. guineensis Jacq*, *E. oleifera* and hybrid fruit bunches in Colombia is 56% of the total FFB weight. We multiplied the annual FFB by 0.56 to obtain the annual supply of oil palm FFB harvested.

# References

Amézquita, E., Rao, I.M., Rivera, M., Corrales, I.I., Bernal, J.H., 2013. Sistemas Agropastoriles: Un Enfoque Integrado para el Manejo Sostenible de Oxisoles de los Llanos Orientales de Colombia. Centro Internacional de Agricultura Tropical (CIAT), Cali.

Arag̃o, L.E.O.C., Malhi, Y., Metcalfe, D.B., Silva-Espejo, J.E., Jiménez, E., Navarrete, D., Almeida, S., Costa, A.C.L., Salinas, N., Phillips, O.L., Anderson, L.O., Alvarez, E., Baker, T.R., Goncalvez, P.H., Huamán-Ovalle, J., Mamani-Solórzano, M., Meir, P., Monteagudo, A., Patĩo, S., Peñuela, M.C., Prieto, A., Quesada, C.A., Rozas-Dávila, A., Rudas, A., Silva Jr, J.A., Vásquez, R., 2009. Above- and below-ground net primary productivity across ten Amazonian forests on contrasting soils. Biogeosciences 6, 2759-2778.

Benavides, J., 2010. El desarrollo económico de la Orinoquia como aprendizaje y construcción de instituciones, Debates Presidenciales. Corporacion Andina de Fomento, CAF, Bogota, Colombia.

Collatz, G.J., Kawa, S.R., 2014. CMS-Flux Pilot Project Land Biosphere Fluxes 2003-2013 from the CASA GFED3 Model, Available online at the North American Carbon Program Website: <http://nacp-files.nacarbon.org/nacp-kawa-01/>

Contreras, B.Á.P., Cayón, S.G., Corchuelo, R.G., 2012. Models to estimate the bunch dry weight in African oil palm (Elaeis guineensis Jacq.), American oil palm (Elaeis oleifera H.B.K. Cortes) and the interspecific hybrid (E. oleifera x E. guineensis). Agronomía Colombiana 30, 46-51.

Corley, R.H.V., Tinker, P., 2008. The oil palm. John Wiley & Sons, Oxford.

FAO, 2010. Global Forest Resources Assessment 2010: Main Report. Food and agriculture organization of the United Nations (FAO), Rome, p. 333.

Fedegan, 2014. Analisis del inventario ganadero Colombiano 2014. Federacion Colombiana de Ganaderos (Fedegan), Colombia.

Fedepalma, 2015. Mini anuario estadistico 2015; principales cifras de la agroindustria de la palma de aceite en Colombia in: Fedepalma (Ed.). Federación Nacional de Cultivadores de Palma de Aceite (Fedepalma), Colombia, p. 64.

Gao, Y.Z., Giese, M., Lin, S., Sattelmacher, B., Zhao, Y., Brueck, H., 2008. Belowground net primary productivity and biomass allocation of a grassland in Inner Mongolia is affected by grazing intensity. Plant. Soil. 307, 41-50.

Gaviria-Uribe, X., Naranjo-Ramírez, J.F., Bolívar-Vergara, D.M., Barahona-Rosales, R., 2015. Intake and digestibility of nutrients in zebu steers grazing in intensive silvopastoral system. Arch. Zootec. 64, 21-27.

Gilmanov, T.G., Parton, W.J., Ojima, D.S., 1997. Testing the ‘CENTURY’ ecosystem level model on data sets from eight grassland sites in the former USSR representing a wide climatic/soil gradient. Ecol. Model. 96, 191-210.

Grigera, G., Oesterheld, M., Pacín, F., 2007. Monitoring forage production for farmers' decision making. Agricultural Systems 94, 637-648.

Gutiérrez-Vélez, V.H., DeFries, R., 2013. Annual multi-resolution detection of land cover conversion to oil palm in the Peruvian Amazon. Remote Sens. Environ. 129, 154-167.

Hui, D., Jackson, R.B., 2006. Geographical and interannual variability in biomass partitioning in grassland ecosystems: a synthesis of field data. New Phytol. 169, 85-93.

Ideam, 2011. Boletin forestal 2008-2010. Instituto de Hidrología, Meteorología y Estudios Ambientales de Colombia (Ideam), Bogota, p. 175.

Kotowska, M.M., Leuschner, C., Triadiati, T., Meriem, S., Hertel, D., 2015. Quantifying above‐and belowground biomass carbon loss with forest conversion in tropical lowlands of Sumatra (Indonesia). Glob. Chang. Biol. 21, 3620-3634.

Lamade, E., Bouillet, J.P., 2005. Carbon storage and global change: The role of oil palm. OCL - Oleagineux Corps Gras Lipides 12, 154-160.

Lasso, C.A., Usma, J.S., Trujillo, F., Rial, B., 2010. Biodiversidad de la cuenca del orinoco: bases científicas para la identificación de áreas prioritarias para la conversación y uso sostenible de la biodiversidad. Instituto de Investigacion de Recursos Biologicos Alexander von Humboldt, Bogotá, D. C., Colombia, p. 304.

López-Mársico, L., Altesor, A., Oyarzabal, M., Baldassini, P., Paruelo, J.M., 2015. Grazing increases below-ground biomass and net primary production in a temperate grassland. Plant. Soil. 392, 155-162.

Lorenz, K., 2013. Ecosystem Carbon Sequestration, in: Lal, R., Lorenz, K., Hüttl, R.F., Schneider, B.U., von Braun, J. (Eds.), Ecosystem Services and Carbon Sequestration in the Biosphere. Springer, Dordrecht, pp. 39-62.

Malhi, Y., Aragão, L.E.O.C., Metcalfe, D.B., Paiva, R., Quesada, C.A., Almeida, S., Anderson, L., Brando, P., Chambers, J.Q., da Costa, A.C.L., Hutyra, L.R., Oliveira, P., Patiño, S., Pyle, E.H., Robertson, A.L., Teixeira, L.M., 2009. Comprehensive assessment of carbon productivity, allocation and storage in three Amazonian forests. Glob. Chang. Biol. 15, 1255-1274.

Malhi, Y., Doughty, C., Galbraith, D., 2011. The allocation of ecosystem net primary productivity in tropical forests. Philos. Trans. R. Soc. Lond. B Biol. Sci. 366, 3225-3245.

Mejía, J., 2000. Consumo de agua por la palma de aceite y efectos del riego sobre la producción de racimos: una revisión de literatura. Revista Palmas 21, 51-58.

Melling, L., Goh, K.J., Beauvais, C., Hatano, R., 2008. Carbon flow and budget in young mature oil palm agroecosystem on deep tropical peat. The Planter 84, 21.

Muñoz, J.M.O., Carvajal, B.N.B., 2013. Analísis de sinergias entre la aplicación de las leyes, la gobernanza y el comercio forestal e iniciativas afines en Brasil, Colombia, Ecuador y Perú. TRAFFIC Internacional, Cambridge, p. 100.

Naidoo, R., Balmford, A., Costanza, R., Fisher, B., Green, R.E., Lehner, B., Malcolm, T.R., Ricketts, T.H., 2008. Global mapping of ecosystem services and conservation priorities. Proc. Natl. Acad. Sci. U.S.A. 105, 9495-9500.

Narváez, J., Chilito, L.A., Bastidas, S., 1996. Determinación de la madurez óptima de cosecha para la palma de aceite (Elaeis guineensis Jacq.) en el región de Tumaco, Nariño. Revista Palmas 17, 15-21.

Oliver, R., 2013. Evaluation and scoping of EU timber importers and imports from South America. TRAFFIC International, Cambridge.

Ott, L.E., Pawson, S., Collatz, G.J., Gregg, W.W., Menemenlis, D., Brix, H., Rousseaux, C.S., Bowman, K.W., Liu, J., Eldering, A., 2015. Assessing the magnitude of CO2 flux uncertainty in atmospheric CO2 records using products from NASA's Carbon Monitoring Flux Pilot Project. J.Geophys.Res-Atmos. 120, 734-765.

Pan, Y., Birdsey, R.A., Phillips, O.L., Jackson, R.B., 2013. The structure, distribution, and biomass of the world's forests, Annu. Rev. Ecol. Evol. Syst., pp. 593-622.

Potter, C., Klooster, S., Genovese, V., Hiatt, C., 2013. Forest production predicted from satellite image analysis for the Southeast Asia region. Carbon Balance Manag. 8, 1-6.

Potter, C.S., Klooster, S., Brooks, V., 1999. Interannual Variability in Terrestrial Net Primary Production: Exploration of Trends and Controls on Regional to Global Scales. Ecosystems 2, 36-48.

Potter, C.S., Randerson, J.T., Field, C.B., Matson, P.A., Vitousek, P.M., Mooney, H.A., Klooster, S.A., 1993. Terrestrial ecosystem production: a process model based on global satellite and surface data. Global Biogeochem. Cycles 7, 811-841.

Poulter, B., Frank, D., Ciais, P., Myneni, R.B., Andela, N., Bi, J., Broquet, G., Canadell, J.G., Chevallier, F., Liu, Y.Y., 2014. Contribution of semi-arid ecosystems to interannual variability of the global carbon cycle. Nature 509, 600-603.

Prada, F., Ayala-Díaz, I.M., Delgado, W., Ruiz-Romero, R., Romero, H.M., 2012. Efecto de la maduración del fruto en el contenido y composición química del aceite de tres materiales de palma de aceite (Elaeis guineensis Jacq) cultivados en Colombia. Revista Palmas 33, 25-39.

Romero-Ruiz, M.H., Flantua, S.G.A., Tansey, K., Berrio, J.C., 2012. Landscape transformations in savannas of northern South America: Land use/cover changes since 1987 in the Llanos Orientales of Colombia. Appl. Geogr. 32, 766-776.

Sarmiento, G., Pinillos, M., 2001. Patterns and processes in a seasonally flooded tropical plain: the Apure Llanos, Venezuela. J. Biogeogr. 28, 985-996.

Schröter, M., Barton, D.N., Remme, R.P., Hein, L., 2014. Accounting for capacity and flow of ecosystem services: A conceptual model and a case study for Telemark, Norway. Ecol. Indic. 36, 539-551.

Scurlock, J.M.O., Johnson, K., Olson, R.J., 2002. Estimating net primary productivity from grassland biomass dynamics measurements. Glob. Chang. Biol. 8, 736-753.

Tan, K.P., Kanniah, K.D., Cracknell, A.P., 2012. A review of remote sensing based productivity models and their suitability for studying oil palm productivity in tropical regions. Prog. Phys. Geogr. 36, 655-679.

Tan, K.P., Kanniah, K.D., Cracknell, A.P., 2013. Use of UK-DMC 2 and ALOS PALSAR for studying the age of oil palm trees in southern peninsular Malaysia. Int. J. Remote Sens. 34, 7424-7446.
